# Prospects of Psychosomatic Medicine

**DOI:** 10.1186/1751-0759-3-1

**Published:** 2009-01-22

**Authors:** Gen Komaki, Yoshiya Moriguchi, Tetsuya Ando, Kazuhiro Yoshiuchi, Mutsuhiro Nakao

**Affiliations:** 1 Department of Psychosomatic Research, National Institute of Mental Health, National Center of Neurology and Psychiatry, Tokyo, Japan; 2 Department of Psychology, Boston College, Boston, USA; 3 Department of Stress Sciences and Psychosomatic Medicine, Graduate School of Medicine, The University of Tokyo, Tokyo, Japan; 4 Department of Hygiene and Public Health, Teikyo University School of Medicine, Itabashi, Tokyo, Japan; 5 Division of Psychosomatic Medicine, Teikyo University Hospital, Itabashi, Tokyo, Japan

## Editorial

Looking back on the history of the psychosomatic medicine in Japan, the focus of our scientific research and medical practice was, at first, the elucidation of the psychosomatic correlation between a patient's neurosis and physical complaints. We then moved in the direction of physical disorders – psychosomatic diseases – in which psychosocial factors were closely involved in the onset and course. We have always advocated the importance of a comprehensive medical approach to the whole person for such physical disorders.

We work from the viewpoint that a psychosomatic disease is not present as a particular "mental" illness, but physical disorders of daily life, "e.g., life-style related diseases". Recently, however, because of rapid changes of the social system, including the information-oriented society, psychosomatic diseases as stress-related disease have increased and the importance of psychosomatic medicine/medical care is currently unprecedentedly high.

Elucidation of functional regulation systems, such as the nervous-endocrine- immune system, which relate mind and body, is an important theme in psychosomatic medicine. Similarly, it is also important to elucidate how and what kind of unbalanced or distorted "psycho-behavioral" factors, such inadequate daily life habits and inappropriate stress coping styles, have influenced the susceptibility to and the continuity of the diseases. This further includes socio-cultural approaches that are epidemiological, not solely focused on either the psychological or behavioral aspect. When we turn our eyes to the diagnostic techniques used in psychosomatic medicine, drastic progress has been made, as shown by the recent research in the neuroscientific, genetic, and behavioral sciences. Through empirical experience backed by these lines of scientific evidence, psychosomatic medicine will be able to further our aim of medical practice based on the whole person concept.

Psychosomatic disease is, generally, not readily improved by conventional medical approaches that are solely based on the treatment of the physical aspects. This is because bio/psycho/behavioral matters are strongly bound by their interaction with various social and environmental factors. We hope this online Internet journal of the Japanese Society of Psychosomatic Medicine – BioPsychoSocial Medicine, , will contribute to the continued evolution of our field.

The general meeting of the 49th Japanese Society of Psychosomatic Medicine was held last year in Sapporo (Chairperson Tsukasa Koyama, Vice-chairperson Etsuji Satohisa). "Progress in emotional stress research and psychosomatic medicine" was advocated as a main theme, and two special lectures were given; "Neuroscientific study of affects and stress" by Taketoshi Ono and "Sex and Sexagenarians" by Lorraine Dennerstein. Eight symposiums were as follows; "The front line of mental health in the work environment", "Adolescent psychosomatic disease and an approach from psychosomatic medicine", "Basics and practices of chronic pain", "Brain-gut correlations related with Functional Dyspepsia", "Problems in diagnosis and treatment of depression and their secrets in daily practice", "The front line of research into eating disorders" "Elucidation of psychosomatic diseases by neuroscience", and "Cooperation between physicians and psychologists involved in psychosomatic medical care in the workplace". In addition, there were eleven educational seminars and a special program "Topics of psychosomatic medicine in the clinical areas – Obstetrics and Gynecology, Dermatology, and Dentistry – ". We were encouraged by the active participation of the attendees.

2009 is a turning point in that it has been 50 years since the foundation of the Japanese Psychosomatic Society in 1959. This was a forerunner of the Japanese Society of Psychosomatic Medicine. In commemoration of our establishment, the first joint meeting of the five Japanese psychosomatic medicine societies and a commemorative ceremony will be held in June at the Tokyo International Forum (Yoshihide Nakai, administration meeting chairperson).

This is the first time in our history that these five societies; The Japanese Society of Psychosomatic Obstetrics And Gynecology, The Japanese Society of Psychosomatic Internal Medicine, The Japanese Society of Psychosomatic Dentistry, and The Japanese Society of Psychosomatic Pediatrics will all meet under the leadership of The Japanese Society of Psychosomatic Medicine. The theme will be "Psychosomatic Medicine as Medical Care of The Near Future". We hope to have lively interaction and promote the spread psychosomatic medicine nationwide. It is our hope that psychosomatic medicine will develop further as a foundation of medicine and medical practice throughout Japan and the world.

Herein, I will introduce trends of the past few years in research fields; such as brain function imaging study, genetic study, behavior science, and epidemiologic study; into psychosomatic medicine, from point-of-view of methods as the basis of clinical medicine and medical care.

## Brain function imaging study

Rapid progress in scientific technology has enabled the non-invasive neuronal imaging of human brain function. Especially, in recent years a staggering increase in functional magnetic resonance imaging (fMRI) study has been seen. fMRI is a method that is comparatively easy and non-invasive, and its spacial resolution is superior to conventional nuclear medicine technologies, such as positron emission tomography (PET), single photon emission computed tomography (SPECT), and electroencephalogram (EEG).

### Diversification of the modality

Trials of combinations of techniques to determine brain activity have begun to emerge [1]: These do not solely measure a specific brain function but at the same time measure various physiological indexes of the autonomic nervous system, such as pulse/skin conductance/electromyogram, to detect dynamic associations of the bodily states with neural states. EEG has been used simultaneously with other neruoimaging techniques like fMRI, to utilize the advantages of the high spatial resolution of MRI and temporal resolution of EEG. The transcranial magnetic stimulation method (TMS) is also used with fMRI as a non-invasive method to temporarily suppress the neuronal activity in a local region by electric stimulation by a coil outside the head. The current trend in neuroscience to focus on brain function as one component of a unified physical system, including the connection between the bodily states and brain activity, seems extremely well matched to the direction of psychosomatic medicine. Furthermore, neuroimaging studies have been done that compare psychosomatic patients and healthy controls and that focus on the association of neuronal states with individual differences in personality tendencies (e.g., alexithymia associated with psychosomatic disorders) [2-5].

### The diversification of the context

Furthermore, there is a new direction of study that includes a wider context than that of specific studies focusing only on a certain disease or nervous system. For example, a new field called 'social neuroscience' is spreading rapidly in which neuroscience has begun to be applied to probing highly advanced cognitive functions, such as what kind of role a neuronal system plays in human social interactions [6,7]. Thus, in the future, neurosciences may also be applied to many issues of the mind-body correlation advocated by psychosomatic medicine, especially to the highly social level of issues in the bio-psycho-social model, that are conventionally difficult to be addressed by the neurosciences, for instance interpersonal relationships.

### Cerebral localization and networks

Recently, a movement has arisen that attempts to understand the function of the brain both comprehensively and integratively as a network [8]: This evolved from the point-of-view that the various sites of the brain and the wide variety of information from the body dynamically form a complicated web of consciousness, recognition, and feelings in a mutually influenced manner.

### Recent study achievements

Some studies using a unique technique called "real time fMRI" (rtfMRI) have been recently reported [9-12]. This method resembles the "biofeedback" therapy that has conventionally been used in psychosomatic medicine to therapeutically give patients feedback on their distal physiological signs. We can call rtfMRI "neurofeedback" in which we use fMRI simultaneously feeding back to the subjects the regional neural activity of the brain so that they can learn to directly control activation of localized regions by themselves -self-control. For example, when subjects were trained and succeeded in achieving voluntary regulation of their right Brodmann's area (BA) 45, extremely interesting results were found; the regional activity increased and a significant improvement of accuracy was observed for the identification of emotional prosodic intonation [12]. Based on these lines of evidence, rtfMRI shows possibilities for use in rehabilitation through training by non-invasive and non-pharmacological means. A trial has just started into the clinical applications for chronic pain/drug dependency/depression and the ability to support psychotherapy [9]. These trials seem quite promising for future use in the field of psychosomatic medicine.

### Conclusion

As research on the brain has advanced, we have found that brain function is even more complicated than previously thought. At the moment there are no clinical applications immediately available, but in the near future the progress of neuroscience shows promise for its clinical utility.

## Genetic research

In psychosomatic medicine, we take into great account the individuality of our patients. A wide variety of human characteristics and individual differences are provided by the environment and heredity. "Human Genetic Variation" was selected as the breakthrough of the year for 2007 by Science [13].

A new era of genome-wide association studies (GWAS) has come and is resulting in systematic, well-powered, genome-wide surveys for the identification of the risk SNPs for complex diseases in the DNA sequence of the human genome. Study has been facilitated by the international HapMap project, equipped by the progress of microarray technology, which has enabled the faster and cheaper examination of millions of SNPs. Associated genes have, in large scale studies, been successively identified for multifactorial diseases such as type II diabetes mellitus [14].

Thus, genes and their function associated with individual differences in susceptibility, variety of symptom, subtype, course, and reactivity to a treatment can be elucidated. This will shed light on factors that will help elucidate the biomedical mechanism(s) of the mind-body relationships

Genetic studies of the important, but common, diseases in the area of psychosomatic medicine, including eating disorders [15] and irritable bowel syndrome [16], are now in progress, and related genes will be identified in the near future.

Genetic elucidation in relation to the stress responsiveness of the hypothalamic – pituitary-adrenal system [17], the autonomic nervous system, and personality traits is currently being done in the Quantitative Trait Loci (QTL) analysis to determine the gene locus responsible for measurable, quantitative character. Studies from the viewpoint of gene-environment interaction (GxE) have also increased [18]. It is interesting how gene and psychosocial factors are mutually associated and contribute to the onset and the course of physical diseases.

Recently, an increasing number of studies in the area of imaging genetics – the use of anatomical or functional imaging technologies as intermediate phenotype (endophenotype) to evaluate the role of genetic variation – have shed light on the genetic basis as well as biological mechanisms involved in individual differences of cognition and emotion [19].

In the following sections we highlight several reports of genetic research done in the field of psychosomatic medicine between the autumns of 2007 and 2008.

### Eating disorders

An association between ED and polymorphism of the brain-derived neurotrophic factor (BDNF) gene was reported [15]. Mercader J M et al. examined transmission disequilibrium with 371 sets of ED trios and parents in a study of 151 Tag SNPs covering ten neurotrophin signaling genes, including BDNF and its receptors, and found significant association with the SNPs of the receptor *NTRK3 *genes among ED patients. Individuals carrying this SNP showed higher levels of *NTRK3 *expression in lymphoblastoid cell lines. Furthermore, higher expression of the orthologous murine *Ntrkk3 *gene was detected in the hypothalamus of an *anx*/*anx *mouse model of anorexia, showing synergetic epistatic interaction with the mutation of the NGFB gene. These lines of evidence suggested a certain role of the point neurotrophin signaling genes to the susceptibility factors for EDs [20].

### Irritable bowel syndrome

The 5-HT3s serotonin receptor type 3 subunit genes are involved in intestinal tract motor – sensory function, and the 5-HT3 receptor antagonist, alosetron, is effective in the treatment of diarrhea predominant IBS (IBS-D). Kapeller J et al. found that the mutation of the untranslated regions (UTRs) of the *HTR3E *serotonin receptor type 3 subunit genes was strongly related to female IBS-D. This is the first example indicating that this mutation modified the adjustment of the gene expression by microRNA [21].

### Gene/environment interaction and stress reactiveness

Increased cardiovascular reactivity (CVR) to mental stress influences the risk of cerebrovascular and heart diseases and their clinical course. Williams RB et al. reported that childhood low socioeconomic status (indexed by Father's Education Level) and the more transcriptionally efficient long (L) allele of a polymorphism of the serotonin transporter gene promoter (5HTTLPR) enhance cardiovascular reactivity to mental stress. However, there was no significant interaction between 5HTTLPR polymorphism and socioeconomic status; both worked additively [22].

### Imaging genetics

Genetically driven variation in neurotransmitter function and brain circuits involved in emotion have attracted our attention [19]. Perlis RH et al. used fMRI and showed that a significant differential activation in insula in response to angry facial expressions is associated with the SNP near the cAMP response element binding protein gene (*CREB1*); the transcriptional control factor gene. CREB has many functions in the regulation of learning and memory, and plays a role in the brain structure related to a reward and evasion. The Perlis group also reported that the same SNP of the *CREB1 *is associated with overt/covert anger expression. It is known that the insula forms a brain network with the amygdale, orbitofrontal cortex, and anterior cingulate gyrus, which have been implicated in processing aversion, such as anger [23].

The role of genetic research in psychosomatic medicine will be much increased in the future. Currently, when we talk about individualized medicine (tailor-made medicine), only genetic information comes to mind. Psychosomatic medicine, however, should aim at the realization of a truly personalized medicine that is based on our comprehension of psychosocial/environmental and genetic factors and their gene-environment interactions.

## Behavioral science

### What is behavioral science?

Behavioral science is considered to be an interdisciplinary study using the methodology of various disciplines. Behavioral science applied to the medical field is behavioral medicine, which is defined as "the interdisciplinary field concerned with the development and integration of psychosocial, behavioral and biomedical knowledge relevant to health and illness and the application of this knowledge to prevention, etiology, diagnosis, treatment and rehabilitation," as stated in the charter of the International Society of Behavioral Medicine (ISBM) (1990). We here introduce distinguished reports on some important topics presented since the autumn of 2007.

### Association between coronary heart diseases and depression

Recently, the association between psychosocial factors and susceptibility/prognosis of coronary heart disease has regained attention [24]. In the autumn of 2007, guidelines on cardiovascular disease (CVD) prevention in clinical practice by nine European societies were presented that consider psychosocial factors as independent risk factors for CVD [25]. In particular, depression (including major depressive disorder as well as depressive symptoms) is regarded as an important risk factor [24]. Several articles have reported "the effects of the treatment of depression on patient outcomes after myocardial infarction ".

### Studies of the effect of pharmacotherapy

At present, there is controversy concerning the effect of antidepressants on patients after myocardial infarction. Although observational secondary analysis on morbidity and mortality in post-myocardial patients who participated in the Enhancing Recovery in Coronary Heart Disease study (ENRICHD) indicated that the use of selective serotonin reuptake inhibitors (SSRI) in depressed patients reduced subsequent fatal cardiovascular event and mortality than non-users [26], the Myocardial INfarction and Depression-Intervention Trial (MIND-IT) showed that SSRI did not give better prognosis in a placebo-controlled trial [27]. However, as shown in the results of the secondary analysis of the MIND-IT [28], patients who were non-responsive to treatment for depression following MI may be associated with subsequent cardiac events.

### Study of the effect of psychotherapy

In the above ENRICHD randomized trial, cognitive behavior therapy (CBT) did not increase event-free survival or reduce subsequent cardiac events compared with the usual care group [29]; however, the secondary analysis of the ENRICHD randomized trial showed that CBT homework adherence could improve depression in the treatment of comorbid depression in those post-MI patients [30].

Furthermore, for depression associated with mortality following MI, a distinguished study was reported in 2008; only new onset depression following myocardial infarction predicts cardiac mortality, but not all depression does so [31]. Thus, the subjects of investigation should be narrowed down in future studies.

### A new clinical assessment method in behavioral science

Recently, ecological momentary assessment (EMA) has been proposed in the behavioral sciences as a reliable method to assess and record events and subjective symptoms as well as physiological and behavioral variables in natural settings. Computerized EMA, i.e. EMA using computers as electronic diaries, has been developed [32] and a symposium on EMA was held at the 10th International Congress of Behavioral Medicine held in August of 2008.

Our group applied EMA using a watch-type computer device for patients with tension-type headache and revealed the importance of assessment of the phenomena at the moment they occur in natural settings [33]. The physical activity level was significantly reduced at the time of headache exacerbation [34].

To explore the future direction of EMA study, it is preferable not only to simply record subjective symptoms but also to record an index of objectivity such as physical activity at the same time, and also to use EMA for intervention studies. Regarding the former direction, our group reported that patients with depression were statistically different from healthy people in terms of the pattern of active period duration with physical activity [35]. By analyzing the behavioral organization of mice with depression experimentally similar to that of humans, we found similar results across species [36]. Regarding the latter, a pilot study using online digital assistance of patients with migraine combined mobile electronic diary monitoring of symptoms with direct online coaching of preventive health behavior [37]. Further development is expected.

## Epidemiologic studies

Several interesting reports have recently been published that add to the epidemiologic findings of stress-related diseases. The final qualifying soccer games of the FIFA World Cup are being held now, in January 2009, and many of you may remember having excitedly watched the 2006 FIFA World Cup games, either in person in Germany or through television. During the one-month period of the last FIFA World Cup, an epidemiological survey was conducted to assess the daily number of patients transported to emergency departments at 15 major German hospitals for the treatment of coronary heart diseases [38]. For the seven matches involving the German team, the daily number of patients visiting the emergency departments for acute coronary heart diseases was significantly increased on six of the seven match days, exclusive of the day when the German team played at the bronze medal match after missing the championship final. The odds ratio of such events on the six days was around 2, when compared to the control period. Thus, it was concluded that cardiovascular events were caused by the excitement of viewing the World Cup soccer games. In lectures on psychosomatic medicine, Japanese students and practitioners have often been presented case studies showing that blood pressure is elevated by watching close Sumo matches and that some hypertensive patients suffer from myocardial infarction because of this excitement. It is interesting that such phenomenon were confirmed epidemiologically in Germany.

Related to the topic of cardiovascular diseases, the Japanese Ministry of Health, Labour, and Welfare, since April, 2008, has required a specific health checkup for patients and persons at high of metabolic syndrome. This checkup covers 57 million people aged 40–74 years old who participate in public health insurance plans, and many enterprises and local government offices are busy to meet the regulations. It is important to clarify if or to what extent mental health conditions are associated with metabolic syndrome and individual metabolic risk factors like obesity and hypertension [39,40]. Because there have been numerous studies examining the type A personality over the past decades, almost all researchers believe there is an association between cardiovascular events and anger/hostility. However, epidemiological reports have just begun to be published showing that metabolic syndrome is associated with depression and anxiety as well. For example, a depressive state, but not anxiety, had a significant, positive association with metabolic syndrome in a study of Japanese male workers [41]. These results will need to be confirmed in further studies.

In the research of psychosomatic medicine, we first should establish reliable and valid quantitative measurements for the assessment of "psychosocial stress" to evaluate quantitatively the effects of psychosocial stressors on the mind/body. In this sense, the maximum or ultimate psychosocial stress is that "a person experiences, witnesses, or is confronted with an event or events that involve actual or threatened death or serious injury, or a threat to the physical integrity of self or others". Post-traumatic stress disorder (PTSD) is an anxiety disorder induced by such an ultimate, psychosocially stressing traumatic event, and it is important to assess PTSD symptoms precisely to understand stress-related disorders. PTSD patients are distressed by clinically characteristic symptoms like nightmares that recall the event for months, hyper vigilance of the autonomic nervous system, and fright reaction and insomnia. However, the psychophysiological mechanism of PTSD still remains unclear. So far, there are several important epidemiological studies of PTSD triggered by recent traumatic events, including the September 11 tragedy, the Iraq War, and man-made and natural disasters, such as large earthquakes in many parts of the world [42-44]. Also, there is an interesting report that prior traumatic experience increases the risk of PTSD after a subsequent trauma [45]. It is important to conduct routine, systematic mental health after exposure to such disasters as great earthquakes and accidents. In addition, we, the researchers, should report evidence of the effectiveness of such systemic interventions done in our clinical practice.

It is a painful experience to face the aftermath of death. Feelings are paralyzed temporarily and bereavement reactions occur when a person loses a close relative or a beloved one [46]. For some people, the depressive state does not recover and lasts for more than a month. It has become a social concern in Japan that the annual number of suicides has been more than 30,000 for the past ten years [47]; consequently, mental health care has become an important issue, not only for the suicide victim but also for the bereaved family. In clinical practice, we must keep a sharp insight into the risks of depression and suicide for patients we see in our daily practice. We must preserve the mind of embrace, carefully listening to a psychologically shocked patient as well [48]. We believe that the accumulation of our steady efforts will result in great power and contribute to the progress of the public health of the whole nation [49].

## Authors' contributions

YM reviewed the recent non-invasive neuronal imaging studies of human brain function. TA reviewed the reports of genetic research done in the field of psychosomatic medicine in recent years. KY introduced the distinguished reports on behavioral science of recent years related to the field of psychosomatic medicine. MN reviewed several interesting studies done over the past few years regarding the epidemiologic findings of stress-related diseases. GK made general remarks on the prospect and future direction of our research and medical practice in psychosomatic medicine and introduced the events of the Japanese Society of Psychosomatic Medicine at present.

All authors read and approved the final manuscript.
